# Supplementary material for: Heat adaptation of phage T7 under an extended genetic code
Source: Virus Evol. 2021 Dec 1;7(2):veab100. doi: 10.1093/ve/veab100 (PMC8923235; doi:10.1093/ve/veab100)
Supplement: veab100_Supp [file veab100_supp.zip › Sequencing_Analysis.zip!/Sequencing_Analysis/S1/evidence/MC_SIDE_2_37.html]

BRESEQ :: Evidence


| Predicted mutation | | | | | | |
| --- | --- | --- | --- | --- | --- | --- |
| evidence | seq id | position | mutation | annotation | gene | description |
| MC JC | NC\_001604.1 | 1,253 | Δ1,489 bp | intergenic (–/–) | – / – | –/– |

| Missing coverage evidence... | | | | | | | | | | |
| --- | --- | --- | --- | --- | --- | --- | --- | --- | --- | --- |
|  |  |  | seq id | start | end | size | ←reads | reads→ | gene | description |
| \* | \* | ÷ | NC\_001604.1 | 1253 | 2741 | 1489 | 4499 [53] | [87] 4530 | *–/–* | –/– |

| New junction evidence | | | | | | | | | | | |
| --- | --- | --- | --- | --- | --- | --- | --- | --- | --- | --- | --- |
|  | | seq id | position | reads (cov) | reads (cov) | score | skew | freq | annotation | gene | product |
| \* | ? | NC\_001604.1 | = 1252 | 53 (0.010) | 4384 (1.080) | 395/580 | 0.0 | 98.5% | intergenic (–/–) | *–/–* | –/– |
|  | ? | NC\_001604.1 | 2742 = | 84 (0.020) |  |  |  |  | intergenic (–/–) | *–/–* | –/– |

Only 100 of 4530 total aligned reads displayed.  

`CAATGATGCCCATTATAAATACGCTGAGATTGCAAGCGACATCATTGATTGCAATTCGGATGAGCATGATGAGTTAACTGGATGGGATGGTGAGTTTGTTGAAACTTGTAAACTAATCCGCAAGTTCTTTGAGGGCATCGCCTCATTCGACATGCATAGCGGGAACATCATGTTCTCAAATGGAGACGTACCATACATCACCGACCCGGTATCATTCTCGCAGAAGAAAGACGGTGGCGCATTCAGCATCGACCCTGAGGAACTCATCAAGGAAGTCGAGGAAGTCGC‑‑ACGACAGAAAGAAATTGACCGCGCTAAGGCCCGTAAAGAACGTCACGAGGGGCGCTTAGAGGCACGCAGATTCAAACGTCGCAACCGCAAGGCACGTAAAGCACACAAAGCTAAGCGCGAAAGAATGCTTGCTGCGTGGCGATGGGCTGAACGTCAAGAACGGCGTAACCATGAGGTAGCTGTAGATGTACTAGGAAGAACCAATAACGCTATGCTCTGGGTCAACATGTTCTCTGGGGACTTTAAGGCGCTTGAGGAACGAATCGCGCTGCACTGGCGTAATGCTGACCGGATGGCTAT  >  NC_001604.1/2455‑3042`  

                                                                                                                                                                                                                                                                                               `|`                                                                                                                                                                                                                                                                                                                
`cgcttacgaaatgctgaaagaaaacatccgttatgatgacatccgtgacactgatgacctgcacgatgctattcacatggctgccgataatgcagttccgcactactacgctgacatctttagcgtaatggcaagtgagggcattgaccttgagttcgaagactctggtctgatgcctgacaccaaggacgtaatccgcatcctgcaagcgcgtatctatgagcaattaacgattgacctctgggaaaacgcagaagacttgctcaatgaatacttggaggaagtcgC‑‑ACGACagaaagaa                                                                                                                                                                                                                                                                                                 >  1:556937‑M2/288‑301 (MQ=255)`  
`cgcttacgaaatgctgaaagaaaacatccgttatgatgacatccgtgacactgatgacctgcacgatgctattcacatggatgccgataatgcagttccgcactactacgctgacatctttagcgtaatggcaagtgagggcattgaccttgagttcgaagaatctggtctggtgcctgacaccaaggacgtaatccgcatcctgcaagcgcgtatctatgagccattaacgattgatctctgggaagacgccgaagacttgctccatgaatccttggaggaagtcgA‑‑ACGACagaaagaa                                                                                                                                                                                                                                                                                                 >  1:299978‑M2/288‑301 (MQ=255)`  
            `gctgaaagaaaacatccgttatgatgacatccgtgacactgatgacctgcacgatgctattcacatggctgccgataatgcagttccgaactactacgctgacatctttagcgtaattgcaagtgagggcattgaccttgagttcgaagactctggtctgatgcctgacaccaatgacgtaatccgcatccttcaagcgcgtatctatgagcaattaccgattgacctctggtaagacgcagaagacttgctcaatgaatacttggaggaagtcgC‑‑ACGACCGAACGAAATTGACCGCGCt                                                                                                                                                                                                                                                                                     >  1:146762‑M2/276‑301 (MQ=255)`  
                 `gctcggagatgtgtataagagacagccgtgacactgatgacctgcacgatgctattcacatggctgccgataatgcagttccgcactactacgctgacatctttagcgtaatggcaagtgagggcattgaccttgagttcgaagactctggtctgatgcctgacaccaaggacgtaatccgcatcctgcaagcgcgtatctatgagcaattaacgattgacctctgggaagacgcagaagacttgctcaatgaatacttggaggaagtcgC‑‑ACGACAGAAAGAAATTGACCGCGCTAAGGc                                                                                                                                                                                                                                                                                <  1:28927‑M2/31‑1 (MQ=255)`  
                                   `atgacatccgtgacactgatgacctgcacgatgctattcacatggctgccgataatgcagttccgcactactacgctgacatctttagcgtaatggcaagtgagggcattgaccttgagttcgaagactctggtctgatgcctgacaccaaggacgtaatccgcatcctgcaagcgcgtatctatgagcaattaacgattgacctctgggaagacgcagaagacttgctcaatgaatacttggaggaagtcgC‑‑ACGACAGAAAGAAATTGACCGCGCTAAGGCCCGTAAAGAACGTCACGa                                                                                                                                                                                                                                                              >  1:306019‑M2/253‑301 (MQ=255)`  
                                     `gacactgatgaccttcacgattctattcacatggctgccgataatgcagttccgcactactacgctgacatctttagcgtaatggcaagtgagggcattgaccttgagttcgaagactctggtcttatgcctgacaccaaggacgtaatccgcatcctgcaagcgcgtatctatgagcaattaacgattgacctctgggaagacgcagaagacttgctcaatgaatacttggaggaagtcgaggaagtcgC‑‑ACGACAGAAAGAAATTGACCGCGCTAAGGCCCGTAAAGAACGTCACGAgg                                                                                                                                                                                                                                                            <  1:19496‑M2/51‑1 (MQ=255)`  
                                       `cactgatgacctgtacgatgctattcacatggctgccgataatgcagttccgcactactacgctgacatctttagcgtaatggcaagtgagggcattgaccttgagttcgaagactctggtctgatgcctgacaccaaggacgtaatccgcatcctgcaatcgcgtatctatgagcaattaacgattgacctctgggaagacgcagaagacttgctcaatgaatacttggaggaagtcgaggaagtcgC‑‑ACGACAGAAAGAAATtgaccgcgctaaggcctgtctcttatacacatctccg                                                                                                                                                                                                                                                          >  1:42446‑M2/249‑283 (MQ=255)`  
                                               `acactgatgacctgcacgatgctattcacatggctgccgataatgcagttccgcactactacgctgacatctttagcgtaatggcaagtgagggcattgactttgagttcgaagactctggtctgatgcctgacaccaaggacgtaatccgcatcctgcaagcgcgtatctatgagcaattaacgattgacctctgggaagacgcagaagccttgctcaatgaatacttggaggaagtcgC‑‑ACGACAGAAAGAAATTGACCGCGATAAGGCCCGTAAAGCACGTCACGAGGGGCGCTTaga                                                                                                                                                                                                                                                  >  1:100235‑M2/241‑301 (MQ=255)`  
                                                 `ctgcacgatgctattcacatggctgccgataatgcagttccgcactactacgctgacatctttagcgtaatggcaagtgagggcattgaccttgagttcgaagactctggtctgatgcctgacaccaaggacgtaatccgcatcctgcaagcgcgtatctatgagcaattaacgattgacctctgggaagacgcagaagacttgctcaatgaatacttggaggaagtcgaggaagtcgC‑‑ACGACAGAAAGAAATTGACCGCGCTAAGGCCCGTAAAGAACGTCACGAGGGGCGCTTAGAgg                                                                                                                                                                                                                                                <  1:32245‑M2/63‑1 (MQ=255)`  
                                                  `ctgatgacctgcacgatgctattcacatggctgccgataatgcagttccgcactactacgctgacatctttagcgtaatggcaagtgagggcattgaccttgagttcgaagactctggtctgatgcctgacaccaaggacgtaatccgcatcctgcaagcgcgtatctatgagcaattaacgattgacctctgggaagacgcagaagacttgctcaatgaatacttggaggaagtcgC‑‑ACGACAGAAAGAAATTTACCGCGCTAAGGCCCGTAAAGAACGTCACGAGGGGCGCTTAGAGGc                                                                                                                                                                                                                                               >  1:48180‑M2/238‑301 (MQ=255)`  
                                                        `acctgcacgatgctattcacatggctgccgataatgcagttccggactactacgctgacatctttagcgtaatggcaagtgagggcattgaccttgagttcgaagactctggtctgatgcctgacaccaatgacgtaatccgcatcctgcaagcgcgtatctatgagcaattaacgattgacctctgggaagacgcagaagacttgctcaatgaatacttggaggaagtcgC‑‑ACGACAGAAAGAAATTGACCGCGCTAAGGCCCGTAAAGAACGTCACGAGGGGCGCTTAGAGGCACGCAg                                                                                                                                                                                                                                         <  1:647955‑M2/70‑1 (MQ=255)`  
                                                            `gcacgatgctattcacatggctgccgataatgcagttccgcactactacgctgacatctttagcgtaatggcaagtgagggcattgaccttgagttcgaagactctggtctgatgcctgacaccaaggacgtaatccgcatcctgcaagcgcgtatctatgagcaattaacgattgacctctgggaagacgcagaagacttgctcaatgaatacttggaggaagtcgC‑‑ACGACAGAAAGAAATTGACCGCGCTAAGGCCCGTAAAGAACGTCACGAGGGGCGCTTAGAGGCACGCAGATTc                                                                                                                                                                                                                                     <  1:506274‑M2/74‑1 (MQ=255)`  
                                                                `agagacagtcaaatggctgccgataatgcagttccgcactactacgctgacatcttgagcgtaatggcaagtgagggcattgaccttgagttcgaagactctggtctgatgcctgacaccaaggacgtaatccgcatcctgcaagcgcgtatctatgagcaattaacgattgacctctgggaagacgcagaagacttgctcaatgaatacttggaggaagtcgC‑‑ACGACAGAAAGAAATTGACCGCGCTAAGGCCCGTAAAGAACGTCACGAGGGGCGCTTAGAGGCACGCAGATTCAAAc                                                                                                                                                                                                                                 <  1:18263‑M2/78‑1 (MQ=255)`  
                                                                 `atgctattcacatggctgccgataatgcagttccgcactactacgctgacatctttagcgtaatggcaagtgagggcattgaccttgagttctaagactctggtctgatgcctgacaccaaggacgtaatccgcatcctgcaagcgcgtatctatgagcaattaacgattgacctctgggaagacgcagaagacttgctcaatgaatacttggaggaagtcgC‑‑ACGACAGAAAGACATTGACCGCGCTAAGGCCCGTAAAGAACGTCACGAGGGGCGCGTAGAGGCACGCAGattcacaag                                                                                                                                                                                                                                >  1:311893‑M2/223‑297 (MQ=255)`  
                                                                      `attcacatggctgccgatactgcagttccgcactactacgctgacatctttagcgtaatggcaagtgagggcattgaccttgagttcgaagactctggtctgatgcctgacaccaaggacgtaatccgcatcctgcaagcgcgtatctatgagcaattaacgattgacctctgggaagacgcagaagacttgctcaatgaatacttggaggaagtcgC‑‑ACGACAGAAAGAAATTGACCGCGCTAAGGCCCGTAAAGAACGTCACGAGGGGCGCTTAGAGGCACGCAGATTCAAACGTCGCa                                                                                                                                                                                                                           >  1:316916‑M2/218‑301 (MQ=255)`  
                                                                                         `atgcagttccgcactactacgctgacatctttagcgtaatggcaagtgagggcattgaccttgagttcgaaggctctggtctgatgcctgacaccaaggacgtaatccgcatcctgcaagcgcgtatctatgagcaattaacgattgacctctgggaagacgcagaagacttgctcaatgaatacttggaggaagtcgC‑‑ACGACAGAAAGAAATTGACCGCGCTAAGGCCCGTAAAGAACGTCACGAGGGGCGCTTAGAGGCACGCAGATTCAAACGTCGCAACCGCAAGGCACGTAAAGc                                                                                                                                                                                                        >  1:353564‑M2/199‑301 (MQ=255)`  
                                                                                                 `ccgcactactacgctgacatctttagcgtaatggcaagtgagggcattgaccttgagttcgaagactctggtctgatgcctgacaccaaggacgtaatccgcatcctgcaagcgcgtatctatgagcaattaacgattgacctctgggaagacgcagaagacttgctcaatgaatacttggaggaagtcgC‑‑ACGACAGAAAGAAATTGACCGCGCTAAGGCCCGTAAAGAACGTCACGAGGGGCGCTTAGAGGCACGCAGATTCAAACGTCGCAACCGCAAGGCACGTAAAGCACACAAAg                                                                                                                                                                                                >  1:258445‑M2/191‑301 (MQ=255)`  
                                                                                                  `acagctactacgcggacatttgtagcgtaatggcaagtgagggcattgaccttgagttcgaagactctggtctgatgcctgacaccaaggacgtaatccgcatcctgcaagcgcgtatctatgagcaattaacgattgaccgctgggaagacgcagaagacttgctcaatgaatacttggaggaagtcgC‑‑ACGACAGAAAGAAATTGACCGCGCTAAGGCCCGTAAAGAACGTCACGAGGGGCGCTTAGAGGCACGCAGATTCAAACGTCGCAACCGCAAGGCACGTAAAGCACACAAAGc                                                                                                                                                                                               <  1:76140‑M2/112‑1 (MQ=255)`  
                                                                                                   `aagagacatcgttgccacctttagcgtaatggcaagtgagggcattgacctttagttcgaagactctggtctgatgcctgacaccaaggacgtaatccgcatcctgcaagcgcgtatctatgagcaattaacgattgacctctgggaagacgcagaagacttgctcaatgaatacttggaggaagtcgC‑‑ACGAAAGAAAGAAATTGACCGCGCTAAGGCCCGTAAAGAACGTCACGAGGGGCGCTTAGAGGCACGCAGATTCAAACGTCGCAACCGAAAGGCACGTAAAGCACACTAAGCt                                                                                                                                                                                              <  1:70160‑M2/113‑1 (MQ=255)`  
                                                                                                      `ctactacgctgacatctttagcgtaatggcaagtgagggcattgaccttgagttcgaagactctggtctgatgcctgacaccaaggacgtaatccgcatcctgcaagcgcgtatctatgagcaattaacgattgacctctgggacgacgcagaagacttgctcaatgcatacttggaggaagtcgC‑‑ACGACAGAAAGAAATTGACCGCGCTAAGGCCCGTAAAGAACGTCACGAGGGGCGCTTAGAGTCACGCAGATTCAAACGTCGCAACCGCAAGGCACGTAAAGCACACCAAGCTAAg                                                                                                                                                                                           >  1:41431‑M2/186‑301 (MQ=255)`  
                                                                                                      `ctactacgctgacatctttagcgtaatggcaagtgagggcattgaccttgagttcgaagactctggtctgatgcctgacaccaaggacgtaatccgcatcctgcaagcgcgtatctatgagcaattaacgattgacctctgggaagacgcagaagacttgctcattgaatacttggaggaagtcgC‑‑ACGACAGAAAGAAATTGACCGCGCTAAGGCCCGTAAAGAACGTCACGAGGGGCGCTTAGAGGCACGCAGATTCAAACGTCGCAACCGCAAGGCACGTAAAGCACACAAAGCGAAg                                                                                                                                                                                           >  1:592193‑M2/186‑301 (MQ=255)`  
                                                                                                      `ctactacgctgacatctttagcgtaatggcaagtgagggcattgaccttgagttcgaagactctggtctgatgcctgacaccaaggacgtaatccgcatcctgcaagcgcgtatctatgagcaattaacgattgacctctgggaagacgcagaagacttgctcaatgaatacttggaggaagtcgC‑‑ACGACAGAAAGAAATTGACCGCGCTAAGGCCCGTAAAGAACGTCACGAGGGGCGCTTAGAGGCACGCAGATTCAAACGTCGCAACCGCAAGGCACGTAAAGCACACAAACGTAAg                                                                                                                                                                                           >  1:597723‑M2/186‑301 (MQ=255)`  
                                                                                                      `ctactacactgacatctttagcgtaatggcaagtgagggcattgaccttgagttcgaagactctggtctgatgcctgacaccaaggacgtaatccgcatcctgcaagcgcgtatctatgagcaattaacgattgacctctgggaagacgcagaagacttgctcaatgaatacttggaggaagtcgC‑‑ACGACAGAAAGAAATTGACCGCGCTAAGGCCCGTAAAGAACGTCACGAGGGGCGCTTAGAGGCACGCAGATTCAAACGTCGCAACCGCACGGCACGTAAAGCACACAAAGCTAAg                                                                                                                                                                                           >  1:13190‑M2/186‑301 (MQ=255)`  
                                                                                                         `ctacgctgacatctttagcgtaatggcaagtgagggcattgaccttgagttcgaagtctctggtctgatgcctgacaccaaggacgtaatccgcatcctgcaagcgcgtatctatgagcaattaacgattgacctctgggaagacgcagaagacttgctcaatgaatacttggaggaagtcgC‑‑ACGACAGAAAGAAATTGACCGCGCTAAGGCCCGTAAAGAACGTCACGAGGGGCGCTTAGAGGCACGCAGATTCAAACGTCGCAACCGCAAGGCACGTAAAGCACACAAAGCTAAgcgc                                                                                                                                                                                        >  1:419170‑M2/183‑301 (MQ=255)`  
                                                                                                          `tacgctgacatctttagcgtaatggaaagtgagggcattgaccttgagttcgaagacgctggtctgaggcctgacaccaaggacgtaaaccgcatcctgcaagcgcgtatctatgagcaattaacgattgacctctgggaagacgcagaagacttgctcaatgaatacttggaggaagtcgC‑‑ACGACAGAAAGAAATTGACCGCGCTAAGGCCCGTAAAGAACGTCACGAGGGGCGCTTAGAGGCACGCAGATTCAAACGTCGCAACCGCAAGGCACGTAAAGCACACAAAGCTAAGcgcg                                                                                                                                                                                       <  1:252274‑M2/120‑1 (MQ=255)`  
                                                                                                           `tctttagcgtaatggcaagtgagggcattgaccttgagttcgaagactctggtctgatgcctgacaccaaggacgtaatccgcatcctgcaagcgcgtatctatgagcaattaacgattgacctctgggaagacgcagaagacttgctcaatgaatacttggaggaagtcgaggaagtcgC‑‑ACGACAGAAAGAAATTGACCGCGCTAAGGCCCGTAAAGAACGTCACGAGGGGCGCTTAGAGGCACGCAGATTCAAACGTCGCAACAGCAAGGCACGTAAAGAAAACAAAGATAAgcgcgg                                                                                                                                                                                      >  1:394180‑M2/181‑300 (MQ=255)`  
                                                                                                           `acgctgacatctttagcgtaatggcaagtgagggcattgaccttgagttcgaagactctggtctgatgcctgacaccaaggacgtaatccgcatcctgcaagcgcgtatctatgagcaattaacgattgacctctgggaagacgcagaagacttgctcaatgaatacttggaggaagtcgC‑‑ACGACAGAAAGAAATTGACCGCGCTAAGGCCCGTAAAGAACGTCACGAGGGGCGCTTAGAGGCACGCAGATTCAAACGTCGCAACCGCAAGGCACGTAAAGCACACAAAGCTAAGCgaga                                                                                                                                                                                      >  1:198695‑M2/181‑301 (MQ=255)`  
                                                                                                                `gacatctttagcgtaatggcaagtgagggcattgaccttgagttcgaagactctggtctgatgcctgacgccaaggacgtaatccgcatcctgcaagcgcgtatctatgagcaattaacgattgacctctgggaagacgcagaagacttgctcaatgaatacttggaggaagtcgC‑‑ACGACAGAAAGAAATTGACCGCGCTAAGGCCCGTCAAGAACGTCACGAGGGGCGCTTAGAGGCACGCAGATTCAAACGTCGCAACCGCAAGGCACGTAAAGCACACAAAGCTAAGCGCGAAAGaa                                                                                                                                                                                 >  1:634265‑M2/176‑301 (MQ=255)`  
                                                                                                                `gacatctttagcgtaatggcaagtgagggcattgaccttgagttcgaagactctggtctgatgcctgacaccaaggacgtaatccgcatcctgcaagcgcgtatctatgagcaattaacgattgacctctgggaagacgcagaagacttgctcaatgaatacttggaggaagtcgC‑‑ACGACAGAAAGAAATTGACCGCGCTAAGGCCCGTAAAGAACGTCACGAGGGGCGCTTAGAGGCACGCAGATTCAAACGTCGCAACCGCAAGGCACGTAAAGCACACAAGGCTAAGCGCGAAAGaa                                                                                                                                                                                 >  1:359336‑M2/176‑301 (MQ=255)`  
                                                                                                                 `gcgtaatggcaagtgagggcattgaccttgagttcgaagactctggtctgatgcctgacaccaaggacgtaatccgcatcctgcaagcgcgtatctatgagcaattaacgattgacctctgggaagacgcagaagacttgctcaatgaatacttggaggaagtcgaggaagtcgC‑‑ACGACAGAAAGAAATTGACCGCGCCAAGGCCCGTAAAGAACGTCACGAGGGGCGCTTAGAGGCACGCAGATTCAAACGTCGCAACCGCAAGGCCCGTAAAGCACACAAAGCTAAGCGCGAAAgaag                                                                                                                                                                                >  1:363557‑M2/175‑300 (MQ=255)`  
                                                                                                                   `gagacaggcgtaatggcaagtgagggcattgaccttgagttcgaagactctggtctgatgcctgacaccaaggacgtaatccgcatcctgcaagcgcgtatctatgagcaattaacgattgacctctgggaagacgcagaagacttgctcaatgaatacttggaggaagtcgC‑‑ACGACAGAAAGAAATTGACCGCGCTAAGGCCCGTAAAGAACGTCACTAGTGGCGTTTAGAGGCACGCAGATTCAAACGTCGCAACCGCAAGGCACGTAAAGCACACAAAGCTAAGCGCGAAAGAAtgc                                                                                                                                                                              <  1:611802‑M2/129‑1 (MQ=255)`  
                                                                                                                   `atcttaagcgtaatggcaagtgagggcattgaccttgagttcgaagactctggtctgatgcctgacagcaaggacgtaatccgcatcctgcaagcgcgtatctatgagcaattaacgattgacctctgggaagacgcagaagacttgctcaatgaatacttggaggaagtcgC‑‑ACGACAGAAAGAAATTGACCGCGCTAAGGCCCGTAAAGAACGTCACGAGGGGCGCTTAGAGGCACGCAGATTCAAACGTCGCAACCGCAAGGCACGTAAAGCACACAAAGCTAAGCGCGAAAGAAtgc                                                                                                                                                                              <  1:401056‑M2/129‑1 (MQ=255)`  
                                                                                                                     `ctttagcgtaatggcaagtgagggcattgaccttgagttcgaagactctggtctgatgcctgacaccaaggacgtaatccgcatcctgcaagcgcgtatctatgagtaattaacgattgacctctgggaagacgcagcagacttgctcaatgaatacttggaggaagtcgC‑‑ACGACAGAAAGAAATTGACCGCGCTAAGGCCCGTAAAGAACGTCACGAGGGGCGCTTAGAGGCACGCAGATTCAAACGTCGCAACCGCAAGGCACGTAAAGCACACAAAGCTAAGCGCGAAAGAAtgctt                                                                                                                                                                            >  1:282959‑M2/171‑301 (MQ=255)`  
                                                                                                                         `aaggtaatggcaagtgagggcattgaccttgagttcgaagactctggtctgatgcctgacaccaaggacgtaatccgcatccggcaagcgcgtatctatgagcaattaacgattgacctctgggaagacgcagaagacttgctcaatgaatacttggaggaagtcgC‑‑ACGACAGAAAGAAATTGACCGCGCTAAGGCCCGTAAAGAACGTCACGAGGGGCGCTTAGAGGCACGCAGATTCAAACGTCGCAACCGCAAGGCACGTAAAGCACACAAAGCTAAGCGCGAAAGAATGCTtgctg                                                                                                                                                                        <  1:512022‑M2/135‑1 (MQ=255)`  
                                                                                                                          `gcgtaatggcaagtgagggcattgaccttgagttcgaagactctggtctgatgcctgacaccaaggacgtaatccgcatcctgcaagcgcgtatctatgagcaattaacgattgacctctgggaagacgcagaagacttgctaaatgaatacttggaggaagtcgC‑‑ACGACAGAAAGAAATTGACCGCGCTAAGGCCCGTAAAGAACGTCACGAGGGGCGCTTAGAGGCACGCAGATTCAAACGTCGCAACCGCAAGGCACGTAAAGCACACAAAGCTAAGCGCGAAAGAATGCTtgctgc                                                                                                                                                                       >  1:94262‑M2/166‑301 (MQ=255)`  
                                                                                                                             `gtgagtgcattgaccttgagttcgaagactctggtctgatgcctgacaccaaggacgtaatccgcatcctgcaagcgcgtatctatgagcaattaacgattgacctctgggaagacgcagaagacttgctcaatgaatacttggaggaagtcgaggaagtcgC‑‑ACGACAGAAAGAAATTGACCGCGCTAAGGCCCGTAAAGAACGTCACGAGGGGCGCTTAGAGGCACGCAGGTTCAAACGTCGCAACCGCAAGGCACGTAAAGCACACAAACCTAAGCGCGACAGAATGCTTGCTTCGTg                                                                                                                                                                    >  1:564143‑M2/163‑301 (MQ=255)`  
                                                                                                                              `tgagggcattgaccttgagttcgaagactctggtctgatgcctgacaccaaggacgtaatccgcatcctgcaagcgcgtatctatgagcaattaacgattgacctctgggaagacgcagaagacttgctcaatgaatacttggaggaagtcgaggaagtcgC‑‑ACGACAGAAAGAAATTGACCGCGCTAAGGCCCGTAAAGAACGTCACGAGGGGCGCTTAGAGGCACGCAGATTCAAACGTCGCAACCGCAAGGCACGTAAAGCACACAAAGCTAAGCGCGAAAGAATGCTTGATGCGTgg                                                                                                                                                                   >  1:45295‑M2/162‑301 (MQ=255)`  
                                                                                                                                  `ggcattgaccttgagttcgaagactctggtctgatgcctgacaccaaggacgtaatccgcatcctgcaagcgagtatctatgagcaattaacgattgacctctgggaagacgcagaagacttgctcaatgaatacttggaggaagtcgaggaagtcgC‑‑ACGACCGAAAGAAATTGACCGCGCTAAGGCCCGTAAAGAACGTCACGAGGGGCGCTTAGAGGCACGCAGATTCAAACGACGCAACCGCCAGGCACGTAAAGCACACAAAGCTAAGCGCGAACGAATGCTTGCTTCGTGGCGAt                                                                                                                                                               >  1:463364‑M2/158‑301 (MQ=255)`  
                                                                                                                                     `agtgagggcattgaccttgagttcgaagactctggtctgatgcctgacaccaaggacgtaatccgcatcctgcaagcgcgtatctatgagcaattaactattgacctctgggaagacgcagaagacttgctcaatgaatacttggaggaagtcgC‑‑ACGACAGAAAGAAATTGACCGCGCTAAGGCCCGTAAAGAACGTCACGAGGGGCGCTTAGAGGCACGCAGATTCAAACGTCGCAACCGCAAGGCACGTAAAGCACACAAAGCTAAGCGCGAAAGAATGCTTGCTGCGTGGCGATggg                                                                                                                                                            >  1:347771‑M2/155‑301 (MQ=255)`  
                                                                                                                                     `agtgagggcattgaccttgagttcgaagactctggtctgatgcctgacaccaaggacgtaatccgcatcctgcaagcgcgtatctatgagcaattaacgattgacctctgggaagacgcagaagacttgctcaatgaatacttggaggaagtcgC‑‑ACGACAGAAAGAAATTGACCGCGCTAAGGCCCGTAAAGAACGTCACGAGGGGCGCTTAGAGGCACGCAGATTCAAACGTCGCAACCGCAAGGCACGTAAAGCACACAAAGCTAAGCGCGAAAGAATGCTTGCTGCGTGGCGATggg                                                                                                                                                            >  1:275896‑M2/155‑301 (MQ=255)`  
                                                                                                                                           `ggcattgaccttgagttcgaagattctggtctgatgcctgacaccaaggacgtaatccgcatcctgcaatcgcgtatctatgagcaattaacgattgacctctgggaagacgcagaagacttgctcaatgaatacttggaggaagtcgC‑‑ACGACAGAAAGAAATTGACCGCGCTAAGGCCCGTAAAGAACGTCACGAGGGGCGCTTAGAGGCACGCAGATTCAAACGTCGCAACCGCAAGGCACGTAAAGCACACAAAGCTAAGCGCGAAAGAATGCTTGCTGCGTGGCGATGGGCTGAAc                                                                                                                                                      >  1:379701‑M2/149‑301 (MQ=255)`  
                                                                                                                                            `gcattgcccgtgagttctaagactctggtctgatgcctgacaccaaggacgtaatccgcatcctgcaagcgcgtatctatgagcaattaacgattgacctctgggaagacgcagaagacttgctcaatgaatacttggaggaagtcgC‑‑ACGACAGAAAGAAATTGACCGCGCTAAGGCCCGTAAAGAACGTCACGAGGGGCGCTTAGAGGCACGCAGATTCAAACGTCGCAACCGCAAGGCACGTAAAGCACACAAAGCTAAGCGCGAAAGAATGCTTGCTGCGTGGCGATGGGCTGAACg                                                                                                                                                     <  1:445‑M2/154‑1 (MQ=255)`  
                                                                                                                                            `gcattgaccttgagttcgaagactctggtctgatgcctgacaccaaggacgtaatccgcatcctgcaagcgcgtatctatgagcaattaacgattgacctctgggaagacgcagaagacttgctcaatgaatacttggaggaagtcgC‑‑ACGACAGAAAGAAATTGACCGCGCTAAGGCCCGTAAAGAACGTCACGAGGGGCGCTTAGAGGCACGCAGATTCAAACGTCGCAACCGCAAGGCACGTAAAGCACACAAAGCTAAGCACGAAAGAATGCTTGCTGCGTGGCGATGGGCTGAACg                                                                                                                                                     >  1:215224‑M2/148‑301 (MQ=255)`  
                                                                                                                                                 `gaccttgagttcgaagactctggtctgatgcctgacaccaaggacgtaatccgcatcctgcaagcgcgtatctatgagcaattaacgattgacctctgggaagacgcagaagacttgctcaatgaatacttggaggaagtcgC‑‑ACGACAGAAAGAAATTGACCGCGCTAAGGCCCGTAAAGAACGTCACGAGGGGCGCTTAGAGGCACGCAGATTCAAACGTCGCAACCTCAAGGCACGTAAAGCACACAAAGCTAAGCGCGAAAGAAtgcttgctgcgtggcgactgtctcttatacaca                                                                                                                                                >  1:458038‑M2/143‑285 (MQ=255)`  
                                                                                                                                                  `accttgagttcgaagactctggtctgatgcctgacaccaaggacgtaatccgcatcctgcaagcgcgtatctatgagcaattaacgattgacctctgggaagacgcagaagacttgctcaatgaatacttggaggaagtcgC‑‑ACGACAGAAAGAAATTGACCGCGCTAAGGCCCGTAAAGAACGTCACGAGGGGCGCTTAGAGGCACGCAGATTCAAACGTCGCAACCGCAAGGCACGTAAAGCACACAAAGCTAAGCGCGAAAGAATGCTTGCTGCGTGGCGATGGGCTGAACGTCAAGa                                                                                                                                               >  1:571893‑M2/142‑301 (MQ=255)`  
                                                                                                                                                         `ctctggtctgatgcctgacaccaaggacgtaatccgcatcctgcaagcgcgtatctatgagcaattaacgattgacctctgggaagacgcagaagacttgctcaatgaatacttggaggaagtcgaggaagtcgC‑‑ACGACAGAAAGAAATTGACCGCGCTAAGGCCCGTAAAGAACGTCACGAGGGGCGCTTAGAGGCACGCAGATTCAAACGTCGCAACCGCAAGGCACGTAAAGCACACAAAGCTAAGCGCGAAAGAATGCTTGCTGCGTGGCGATGGGCTGAACGTCAAGAACGGCGt                                                                                                                                        >  1:430648‑M2/135‑301 (MQ=255)`  
                                                                                                                                                            `cgatgactctggtctgatgcctgacaccaaggacgtaatccgcatcctgcaagcgcgtatctatgagaaattaacgattgacctctgggaagacgcagaagacttgctcaatgaatacttggaggaagtcgC‑‑ACGACAGAAAGAAATTGACCGCGCTAAGGCCCGTAAAGAGCGTCACGAGGGGCGCTTAGAGGCACGCAGATTCAAACGTCGCAACCGCAAGGCACGTAAAGCACACAAAGCTAAGCGCGAAAGAATGCTTGCTGCGTGGCGATGGGCTGAACGTCAAGAACGGCGTAAc                                                                                                                                     <  1:112280‑M2/170‑1 (MQ=255)`  
                                                                                                                                                              `aagacgctggtctgatgccggaaaccaagaaggtaatccgcatgctgcaagggcgtatctatgagcaattaacgattgacctctgggaagacgcagaagacttgctcaatgaatacttggaggaagtcgC‑‑ACGACAGAAAGAAATTGACCGCGCTAAGGCCCGTAAAGAACGTCACGAGGGGCGCTTAGAGGCACGCAGATTCAAACGTCGCAACCGCAAGGCACGTAAAGCACACAAAGCTAAGCGCGAAAGAATGCTTGCTGCGTGGCGATGGGCTGAACGTCAAGAACGGCGTAACCa                                                                                                                                   <  1:385568‑M2/172‑1 (MQ=255)`  
                                                                                                                                                                    `ctggtctgatgcctgaaaccaaggacgtaatccgcaacctgcaagcgcgtatctatgagcaattaacgattgacctctgggaagacgcagaagacttgctcaatgaatacttggaggaagtcgC‑‑ACGACAGAAAGAAATTGACCGCGCTAAGGCCCGTAAAGAACGTCACGAGGGGCGCTTAGAGGCACGCAGATTCAAACGTCGCAACCGCAAGGCACGTAAAGCACACAAAGCTAAGCGCGAAAGAATGCTTGCTGCGTGGCGATGGGCTGAACGTCAAGAACGGCGTAACCATGAGGt                                                                                                                             <  1:518285‑M2/178‑1 (MQ=255)`  
                                                                                                                                                                          `tgataacagacacaaaggacgtaagccgactcctgcaagcgcgaatcgatgagcaattaacgattgacctcgggaaagacgcagaagacttgctcaatgaatacttggaggaagtcgC‑‑ACGACAGAAAGAAATTGACCGCACTAAGTCCCGTAAAGAACGTCACGAGGGGCGCTTAGAGGCACGCAGATTCAAACGTCGCAACCGCAAGGCACGTAAAGCACACTAAGCTAAGCGCGAAAGAATGCTTGCTGCGTGGCGATGGGCTGAACGTCAAGAACGGCGTAACCATGAGGTAGCTGt                                                                                                                       <  1:623685‑M2/184‑1 (MQ=255)`  
                                                                                                                                                                              `gcctgacaccaaggacgtaatccgcatcctgcaagcgcgtatctatgtgcaattaacgattgacctctgggaagacgcagaagacttgctcaatgaatacttggaggaagtcgC‑‑ACGACAGAAAGAAATTGACCGCGCTAAGGCCCGTAAAGAACGTCACGAGGGGCGCTTAGAGGCACGCAGATTCAAACGTCGCAACCGCAAGGCACGTAAAGCACACAAAGCTAAGCGCGAAAGAATGCTTGCTGCGTGGCGATGGGCTGAACGTCAAGAACGGCGTAACCATGAGGTAGCTGTAGAt                                                                                                                   >  1:654272‑M2/114‑301 (MQ=255)`  
                                                                                                                                                                               `gacagacccaaggacgtaatccgcatcctgcaagcgcggatctatgagcaattaacgattgacctctgggaagacgcagaagacttgctcaatgaatacttggaggaagtcgC‑‑ACGACAGAAAGAAATTGACCGCGCTAAGGCCCGTAAAGAACGTCACGAGGGGCGCTTAGAGGCACGCAGATTCAAACGTCGCAACCGCAAGGCACGTAAAGCACACAAAGCTAAGCGCGAAAGAATGCTTGCTGCGTGGCGATGGGCTGAACGTCAAGAACGGCGTAACCATGAGGTAGCTGTAGATg                                                                                                                  <  1:500103‑M2/189‑1 (MQ=255)`  
                                                                                                                                                                               `cctgccaccaaggacggaatccgcatcctgaacgcgcttatcgaggagcaattaacgattgacctcttggaagaccaagaagaattgctcaatgaatacttggaggaagtcgC‑‑ACGACAGAAAGAAATTGACCGCGCTAAGGCCCGTAAAGAACGTCACGAGGGGCGCTTAGAGGCACGCAGATTCAAACGTCGCAACCGCAAGGCACGTAAAGCACACAAAGCTAAGCGCGAAAGAATGCTTGCTGCGTGGCGATGGGCTGAACGTCAAGAACGGCGTAACCATGAGGTAGCTGTAGATg                                                                                                                  <  1:408016‑M2/189‑1 (MQ=255)`  
                                                                                                                                                                               `cctgacaccaaggacgtaatccgcatcctgcaagcgcgtatctatgagcaattaacgattgacctctgggaagacgcagaagacttgctcaatgaatacttggaggaagtcgC‑‑ACGACAGAAAGAAATTGACCGCGCTAAGGCCCGTAAAGAACGTCACGAGGGGCGCTTAGAGGCACGCAGATTCAAACGTCGCAACCGCAAGGCACGTAAAGCACACAAAGCTAAGCGCGCAAGAATGCTTGCTGCGTGGCGCTGGGCTGAACGTCAAGAACGGCGTAACCCTGAGGTAGCTGTagact                                                                                                                  >  1:643855‑M2/113‑299 (MQ=255)`  
                                                                                                                                                                               `cctgacaccaaggacgtaatccgaatcctgcaagcgcgtatctatgagcaattaacgattgacctctgggaagacgcagaagacttgctcaatgaatacttggaggaagtcgC‑‑ACGACAGAAAGAAATTGACCGCGCTAAGGCCCGTAAAGAACGTCACGAGGGGCGCTTAGAGGCACGCAGATTCAAACGTCGCAACCGCAAGGCACGTAAAGCACACAAAGCTAAGCGCGAAAGAATGCTTGCTGCGTGGCGATGGGCTGAACGTCAAGAACGGCGTAACCATGAGGTAGCTGTAGATg                                                                                                                  <  1:265309‑M2/189‑1 (MQ=255)`  
                                                                                                                                                                               `catgacaccaaggacgtaatccgcatcctgcaagcgcgtatctatgagcaattaacgattgacctctgggaagacgcagaagacttgctcaatgaatacttggaggaagtcgC‑‑ACGACAGAAAGCAATTGACCGCGCTAAGGCCCGTAAAGAACGTCACGAGGGGCGCTTAGAGGCACGCAGATTCAAACGTCGCAACCGCAAGGCACGTAAAGCACACAAAGCTAAGCGCGAAAGAATGCTTGCTGCGTGGCGATGCGCTGAACGTCAAGAACGGCGTAACCATGAGGTAGCTGTAGATg                                                                                                                  <  1:551524‑M2/189‑1 (MQ=255)`  
                                                                                                                                                                                  `cgcaccaaggacgtagtccgcatcctgcaagcgcgtttctatgagcaattaacgattgacctctgggaagacccagaagacttgctcaatgaatacttggaggaagtcgC‑‑ACGACAGAAAGAAATTGACCGCGCTAAGGCCCGTAAAGAACGTCACGAGGGGCGCTTAGAGGCACGCAGATTCAAACGTCGCAACCGCAAGGCACGTAAAGCACACAAAGCTAAGCGCGAAAGAATGCTTGCTTCGTGGCGATGGTCTGAACGTCAAGAACGGCGTAATCATGAGGTAGCTGTAGATGTAc                                                                                                               <  1:125692‑M2/192‑1 (MQ=255)`  
                                                                                                                                                                                    `caccaaggacgtaatccgcatcctgcaagcgcgtatctatgagcacttaacgattgacctctgggaagacgcagaagacttgctcaatgaatacttggaggaagtcgC‑‑ACGACAGAAAGGAATTGACCGCGCTAAGGCCCGTAAAGAACGTCACGAGCGGCGCTTAGAGGCACGCAGATTCAAGCGTCGCAACCGCAAGGCACGCAAAGCACACAAAGCTAAACGCGAAAGAATGCTTGCTGCGTGGCGATGGGCTGAACGTCAAGGACGGCGTAACCATCAGGTATCCGTAGACGTACTa                                                                                                             >  1:210116‑M2/108‑301 (MQ=255)`  
                                                                                                                                                                                    `aaccaaggacgtaatccgcatcctgcaagcgcgtatctatgagcaattaacgattgacctctgggaagacgcagaagacttgctcaatgaatacttggaggaagtcgC‑‑ACGACAGAAAGAAATTGACCGCGCTAAGGCCCGTAAAGAACGTCACGAGGGGCGCTTAGAGGCACGCAGATTCAAACGTCGCAACCGCAAGGCACGTAAAGCACACAAAGCTAAGCGCGAAAGAATGCTTGCTGCGTGGCGATGGGCTGAACGTCAAGAACGGCGTAACCATGAGGTAGCTGTAGATGTACTa                                                                                                             <  1:639368‑M2/194‑1 (MQ=255)`  
                                                                                                                                                                                    `aaccaaggacgtaatccgcatcctgcaagcgcgtatctatgagcaattaacgattgacctctgggaagacgcagaagacttgcgaaatgaatacttggaggaagtcgC‑‑ACGAAAGAAAGAAATTGACCGCGCTAAGGCCCGTAAAGAACGTCACGAGGGGCGCTTAGAGGCACGCAGATTCAAACGTCGCAACCGCAAGGCACGTAAAGCACACAAAGCTAAGCGCGAAAGAATGCTTGCTGCGTGGCGATGGGCTGAACGTCAAGAACGGCGTAACCATGAGGTAGCTGTAGATGTACTa                                                                                                             <  1:489436‑M2/194‑1 (MQ=255)`  
                                                                                                                                                                                     `cccaaggacgtaatccgcaccctgcaagcgcgtatctatgagcaattaacgattgacctctgggaagacgcagaagacttgctcaatgaatacttggaggaagtcgC‑‑ACGACAGAAAGAAATTGAACGCGCTAAGGCCCGTAAAGAACGTCACGAGGCGCGCTTAGAGGCACGCAGATTCACACGTCGCAACCGCAAGGCACGTAAAGCACACAAAGCTAAGCGCGAAAGAATGCTTGCTGCGTGGCGATGGGCTGAACGTCAAGAACGGAGTAACCATGAGGTAGCTGTAGATGTACTAg                                                                                                            <  1:524766‑M2/195‑1 (MQ=255)`  
                                                                                                                                                                                       `caaggccgtaatccgcatcctgcaagcgcgtatctatgagcaattaacgattgacctctgggaagacgcagaagacttgctcaatgaatacttggaggaagtcgC‑‑ACGACAGAAAGAAATTGACCGCGCTAAGGCCCGTAAAGAACGTCACGAGGGGCGCTTAGAGGCACGCAGATTCAAACGTCGCAACCGCAAGGCACGTAAAGCACACAAAGCTAAGCGCGAAAGAATGCTTGCTGCGTGGCGATGGGCTGAACGTCAAGAACGGCGTAACCATGAGGTAGCTGTAGATGTACTAGga                                                                                                          <  1:32923‑M2/197‑1 (MQ=255)`  
                                                                                                                                                                                       `caaggacgtaatccgcatcctgcaagcgagtatcgatgggcaattaacgagtgaactatgggaagacgcagaagaagtgctgaatgaataattggaggaagtcgC‑‑ACGACAGAAAGAAATTGACCGCGCTAAGGCCCGTAAAGAACGTCACGAGGGGCGCTTAGAGGCACGCAGATTAAAACGTCGCAACCGCAAGGCACGGAAAGCAAACAAAGCTAAGTGCGAAAGAATGCTTGCTGCGTGGCGATGGGATGAACGTCAAGAACGGCGTAACCATGAGGTAGCTGTAGATGTACTAGga                                                                                                          <  1:240683‑M2/197‑1 (MQ=255)`  
                                                                                                                                                                                       `agagacagtcctgcaagcgcgtatctaggagaaattaacgagtgacctcggagaagacgcagaagaattgatcaatggatacttggaggaagtagaggaagtcgC‑‑ACAACAGAAAGAAATTAACCGCGCAAAGGCCCGTAAAGAACGTCACGAGGGGCGATTAGAGGCACGCAGATTCAAACGTCGCAACCGCAAGGCACGTAAAGCACACAAAGCTAAGCGCGAAAGAATGCTTGCTGCGTGGCTATTGGCTGAACGTCAAGAACGGCGTAACCATGAGGTAGCTGTAGATGTACTAGga                                                                                                          <  1:228179‑M2/197‑1 (MQ=255)`  
                                                                                                                                                                                       `aatccgcatcctgcaagcgcgtatctatgagcaattaacgattgacctctgggaagccgcagaagacctgctcaatgaatgcttttaggaagtcgaggaagtcgC‑‑ACGACAGAAAGAAATTGACCGCGCTAAGGCCCGTAAAGAACGTCACGAGGGGCGCTTAGAGGCACGCAGATTCAAACGTCGCAACCGCAAGGCACGTAAAGCACACAAAGCTAAGCGCGAAAGAATGCTTGCTGCGTGGCGATGGGCTGAACGTCAAGAACGGCGTAACCATGAGGTAGCTGTAGATGTACTAGga                                                                                                          <  1:578744‑M2/197‑1 (MQ=255)`  
                                                                                                                                                                                       `aatccgcatcctgcaagcgcgtatctatgagcaagtaacgattgacctctgggaagacgcagaagacttgctcaatgaatacttggaggaagtcgaggaagtcgC‑‑ACGACAGAAAGAAATTGACCGCGCTAAGGCCCGTAAAGAACGTCACGAGGGGCGCTTAGAGGCACGCAGATTCAAACGTCGCAACCGCAAGGCACGTAAAGCACACAAAGCTAAGCGCGAAAGAATGCTTGCTGCGTGGCGATGGGCTGAACGTCAAGAACGGCGTAACCATGAGGTAGCTGTAGATGTACTAGga                                                                                                          <  1:170705‑M2/197‑1 (MQ=255)`  
                                                                                                                                                                                         `tccgaatcctgcaagcgcgtatctttgagcaattaacgattgacctctgggaagacgcagaaaacttgctaaataaatacttggaggaagtcgaggaagtcgC‑‑ACGACAGAAATAAATTGACCGCGCTAAGGCCCGTAAAGAACGTCACGATGGGCGCGTAGAGGCACGCAGATTCAAACGTCGAAACCGCAAGGCACGTAAAGCACACAAAGCTAAGCGCGAAAGAGTGCTTGCTGCGTGGCGATGGGCTGAACGTCAAGAACGGCGTAACCATGAGGTAGCTGTAGATGTACTAGgaag                                                                                                        <  1:669032‑M2/199‑1 (MQ=255)`  
                                                                                                                                                                                           `gacgtaatccgcatcctgcaagcgcgtatctatgagcaattaacgattgacctctgggaagacgcagaagacttgctcaatgaatacttggaggaagtcgC‑‑ACGACAGAAAGAAATTGACCGCGCTAAGGCCCGTAAAGAACGTCACGAGGGGCGCTTAGAGGCACGCAGATTCAAACGTCGCAACCGCAAGGCACGTAAAGCACACAAAGCTAAGCGCGAAAGAATGCTTGCTGCGTGGCGATGGGCTGAACGTCAAGAACGGCGTAACCATGAGGTAGCTgtagatgtacctgtctctt                                                                                                      >  1:494452‑M2/101‑292 (MQ=255)`  
                                                                                                                                                                                             `cgtaatccgcatcctgcaagcgcgtatctatgagcaattaatgattgacctctgggaagacgcagaagacttgctaaatgaatacttggaggaagtcgC‑‑ACGACAGAAAGAAATTGACCGCGCTAAGGCCCGTAAAGAACGTCACGAGGGGCGCTTAGAGGCACGCAGATTCAAACGTCGCAACCGCAAGGCACGTAAAGCACACAAAGCTAAGCGCGAAAGAATGCTTGCTGCGTGGCGATGGGCTGAACGTCAAGAACGGCGTAACCATGAGGTAGCTGTAGATGTACTAGGAAGAAcc                                                                                                    <  1:270858‑M2/203‑1 (MQ=255)`  
                                                                                                                                                                                             `catcctgcaagcgcgtatctatgagcaattaacgattgacctctgggaagacgcagaagacttgctcaatgaatacttggaggaagtcgaggaagtcgC‑‑ACGACAGAAAGAAATTGACCGCGCTAAGGCCCGTAAAGAACGTCACGAGGGGCGCTTAGAGGCACGCAGATTCAAACGTCGCAACCGCAAGGCACGTAAAGCACACAAAGCTAAGCGCGAAAGAATGCTTGCTGCGTGGCGATGGGCTGAACGTCAAGAACGGCGTAACCATGAGGTAGCTGTAGATGTACTAGGAAGAAcc                                                                                                    <  1:235700‑M2/203‑1 (MQ=255)`  
                                                                                                                                                                                                  `tccgcatcctgcaagcgcgtatctatgagcaattaacgattgacctctgggaagacgcagaagacttgctcaatgaatacttggaggaagtcgC‑‑ACGACAGAAAGAAATTGACCGCGCTAAGGCCAGTAAAGAACGTCACGAGGGGCGCTTAGAGGCACGCAGATTCAAACGTCGCAACCGCAAGGCACGTAAAGCACACAAAGCTAAGCGAGAAAGAATGCTTGCTGCGTGGCGATGGGCTGAACGTCAAGAACGTCGTAACCATGAGGTAGCTGTAGATGTACTAGGAAGAACCAATaa                                                                                               <  1:530513‑M2/208‑1 (MQ=255)`  
                                                                                                                                                                                                  `agcaagcgcgtatctatgagcaattaacgattgacctctgggaagacgcagaagacttgctcaatgaatacttggaggaagtcgaggaagtcgC‑‑ACGACAGAAAGAAATTGACCGCGCTAAGGCCCGTAAAGAACGTCACGAGGGGCGCTTAGAGGCACGCAGATTCAAACGTCGCAACCGCAAGGCACGTAAAGCACACAAAGCTAAGCGCGAAAGAATGCTTGCTGCGTGGCGATGGGCTGAACGTCAAGAACGGCGTAACCATGAGGTAGCTGTAGATGTACTAGGAAGAACCAATaa                                                                                               <  1:470279‑M2/208‑1 (MQ=255)`  
                                                                                                                                                                                                   `ccgcatcctgcaagcgcgtatctatgagcaaataacgattgacctctgggaagacgcagaagacttgctcaatgaatacttggaggaagtcgC‑‑ACGAAAGAAAGAAATTGACCGCGCTAAGGCCCGTAAAGAACGTCACGAGGGGCGCTTAGAGGCACGCAGATTCAAACGTCGCAACCGAAAGGCACGTAAAGCACACAAAGCTAAGCGCGAAAGAATGCTTGCTGCGTGGCGATGGGCTGAACGTCAAGAACGGCGTAACCATGAGGTAGCTGTAGATGTACTAGGAAGAACCAATAAc                                                                                              <  1:436214‑M2/209‑1 (MQ=255)`  
                                                                                                                                                                                                     `gcatcctgcaagcgcgtatctatgagcaattaacgattgacctctgggaagacgcagaagacttgctcaatgaatacttggaggaagtcgC‑‑ACGACAGAAAGAAATTGACCGCGCTAAGGCCCGTAAAGAACGTCACGAGGGGCGCTTAGAGGCACGCAGATTCAAACGTCGCAACCGCAAGGCACGTAAAGCACACAAAGCTAAGCGCGAAAGAATGCTTGCTGCGTGGCGATGGGCTGAACGTCAAGAACGGCGTAACCATGAGGTAGCTGTAGATGTACTAGGAAGAACCAATAACGc                                                                                            <  1:87498‑M2/211‑1 (MQ=255)`  
                                                                                                                                                                                                        `acctgcaagcgcgtatctatgagcaattaacgagtgacctctgggaaaacgcagaaggcttgctcaatgaatacttgaaggaagtcgA‑‑ACGACAGAAAGAAATTGACCGCGCTAAGGCCCGTAAAGAACATCACGAGGGGCGCTTAGAGGCACGCAGATTCAAACGTCGCAACCGCAAGGCACGTAAAGCACACAAAGCTAAGCGCGAAAGAATGCTTGCTGCGTGGCGATGGGCTGAACGTCAAGAACGGCGTAACCATGAGGTAGCTGTAGATGTACTAGGAAGAACCAATAACGCTAt                                                                                         <  1:247005‑M2/214‑1 (MQ=255)`  
                                                                                                                                                                                                            `gcaagcgcgtatctatgagcaattaacgattgacctctgggaagacgcagaagacttgctcaatgaatacttggaggaagtcgC‑‑ACGACAGAAAGAAATTGACCGCGCTAAGGCCCGTAAAGAACGTCACGAGGGGCGCTTAGAGGCACGCAGATTCAAACGTCGCAACCGCAAGGCACGTAAAGAACACAAAGCTAAGCGCGAAAGAATGCTTGCTGCGTGGCGATGGGCTGAACGTCAAGAACGGCGTAACCATGAGGTAGCTGTAGATGTACTAGGAAGAACCAATAACGCTATGctc                                                                                     <  1:220941‑M2/218‑1 (MQ=255)`  
                                                                                                                                                                                                            `aaagagacagatctatgagcaattaacgattgacctctgggaagacgcagaagacttgctcaatgaatacttggaggaagtcgC‑‑ACGACAGAAAGAAATTGACCGCGCTAAGGCCCGTAAAGAACGTCACGAGGGGCGCTTAGAGGCACGCAGATTCAAACGTCGCAACCGCAAGGCACGTAAAGCACACAAAGCTAAGCGCGAAAGAATGCTTGCTGCGTGGCGATGGGCTGAACGTCAAGAACGGCGTAACCATGAGGTAGCTGTAGATGTACTAGGAAGAACCAATAACGCTATGctc                                                                                     <  1:545834‑M2/218‑1 (MQ=255)`  
                                                                                                                                                                                                              `tatcaatgagcaattaacgattgacctctgggaagacgaagaagactagctcaatgaatacttggaggaagtcgaggaagtCGAACGACAGAAAGAAATTGACCGCGCTAAGGCCCGTAAAGAACGTCACGAGGGGCGCTTAGAGGCACGCAGATTCAAACGTCGCAACCGCAAGGCACGTAAAGCACACAAAGCTAAGCGCGAAAGAATGCTTGCTGCGTGGCGATGGGCTGAACGTCAAGAACGGCGTAACCATGGGGTAGCTGTAGATGTACTAGGAAGAACCAATAACGCTATGctc                                                                                     <  1:617459‑M2/220‑1 (MQ=255)`  
                                                                                                                                                                                                               `agcgcgtatctatgagcaattaacgattgacctctgggaagacgcagaagacttgctcaatgaatacttggaggaagtcgC‑‑ACGACAGAAAGACATTGACCGCGCTAAGGCCCGTAAAGAACGTCACGAGGGGCGCTTAGAGGCACGCAGCTTCAAACGTCGCAACCGCAAGGCACGTAAAGCACACAAAGCTAAGCGCGAAAGAATACTTGCTGCGTGGCGATGGGCTGAACATCAAGAACGTCTTAACCATGAGGTAGCTGTAGCTGTCCTAGGAAGAACCCATAACGCTCTGCTCTgg                                                                                  >  1:476086‑M2/81‑301 (MQ=255)`  
                                                                                                                                                                                                                 `atgagcaattaacgagtgacctctgggaagacgcagaagacttgctcaatgaatacttggaggaaggcgaggaagtcgC‑‑ACGACAGAAAGAAATTGACCGCGCTAAGGCCAGTAAAGAACGTCACGAGGGGCGCGTAGAGGCACGCAGATGCAAACGTCGCAACCGCAAGGCACGTAAAGCACACAAAGCGAAGCGCGAAAGAATGCTTGCTGCGTGGCGAGGGGCTGAACGGCAAGAACGGCGTAACCAGGAGGTAGCTGTAGATGTACTAGGAAGAACCAATAACGCTATGCTCTGGGt                                                                                <  1:164463‑M2/223‑1 (MQ=255)`  
                                                                                                                                                                                                                             `cgattgaccgctgggaagacgcagaagacttgctcaatgaatacttggaggaagtcgaggaagtcgC‑‑ACGACAGAAAGAAATTGACCGCGCTAAGGCCCGTAAAGAACGTCACGAGGGGCGCTTAGAGGCACGCAGATTCAAACGTCGCAACCGCAAGGCACGTAAAGCACACAAAGCTAAGCGCGAAAGAATGCTTGCTGCGTGGCGATGGGCTGAACGTCAAGAACGGCGTAACCATGAGGTAGCTGTAGATGTACTAGGAAGAACCAATAACGCTATGCTCTGGGTCAACATGTtctc                                                                    <  1:658772‑M2/235‑1 (MQ=255)`  
                                                                                                                                                                                                                             `agcaattaatgattgacctgtgggaagacgcagaagacttgctcaatgaatacttggaggaagtcgC‑‑ACGACAGAAAGAAATTGACCGCACTAAGGCCCGTAAAGAACGCCACGAGGGGCGCTTAGAGGCACGCAGATTCAAACGTCGCAACCGCAAGGCACGTAAAGCACACAAAGCTAAGCGCGAAAGAATGCTTGCTGCGTGGCGATGGGCTGAACGTCAAGAACGGCGTAACCATGAGGTAGCTGTAGATGTACTAGGAAGAACCAATAACGCTATGCTCTGGGTCAACATGTtctc                                                                    <  1:176292‑M2/235‑1 (MQ=255)`  
                                                                                                                                                                                                                              `gcaattaacgattgacctctgggaagacgcagaagacttgctcaatgaatacttggaggaagtcgC‑‑ACGACAGAAAGAAATTGACCGCGCTAAGGCCCGTAAAGAACGTCACGAGGGGCGCTTAGAGGTACGCAGATTCAAACGTCGCAACCGCAAGGCACGTAAAGCACACAAAGCTAAGCGCGAACGACTGCTTGCTGCGTGGCGATGGGCTGAACGTCAAGAACGGCGTAACCATGAGGTAGCTGTAGATGTATTAGGAAGAACCAATAACGCTATGCTCTGGGTCAACATGTCctct                                                                   >  1:158460‑M2/66‑301 (MQ=255)`  
                                                                                                                                                                                                                                 `attaacgattgacctctgggaagacgcagaagacttgctcaatgaatacttggaggaagtcgC‑‑ACGACAGAAAGAAATTTACCGCGCTAAGGCCCGTAAAGAACGTCACGAGGGGCGCTTAGAGGCACGCAGATTCAAACGTCGCAACCGCAAGGCACGTAAAGCACACAAAGCTAAGCGCGAAAGAATGCTTGCTGCGTGGCGATGGGCTGAACGTCAAGAACGGCGTAACCATGAGGTAGCTGTAGATGTACTAGGAAGAACcaataacgctatgctctgggctgtctcttatacacat                                                                >  1:62905‑M2/63‑284 (MQ=255)`  
                                                                                                                                                                                                                                 `attaacgattgacctctgggaagacgcagaagacttgctcaatgaatacttggaggaagtcgC‑‑ACGACAGAAAGAAATTGACCGCGCTAAGGCCCGTCAAGAACGTCACGAGGGGCGCTTAGAGGCACGCAGATTCACTCGTCGCAACCCCAAGGCACGTAAAGCACACAAAGCTAAGCGCGAAAGAATGCTTGCTGCGTGGCGATGGGCTGAACGTCAAGAACGGCGTAAGCATGAGGTAGCTGTAGATGTACTAGGAAGAACCAATAACGCTATGCTCTGGACCCACATTTTCTCTggg                                                                >  1:664442‑M2/63‑301 (MQ=255)`  
                                                                                                                                                                                                                                  `ttaacgattgacctctgggaagacgcagaagacttgctcaatgaatacttggaggaagtcgC‑‑ACGACAGAAAGAAAGTGACCGCGCTAAGGCCCGTAAAGAACGTCACGAGGGGCGCTTAGAGGCACGCAGATTCAAACGTCGCAACCGCAAGGCACGTAAAGCACACAAAGCTAAGCGCGAAAGAATGCTTGCTGCGTGGCGATGGGCTGAACGTCAAGAACGGCGTAACCATGAGGTAGCTGTAGATGTACTAGGAAGAACCAATAACGCTATGCTCTGGGTCAACATGTTCTCTgggg                                                               <  1:10707‑M2/240‑1 (MQ=255)`  
                                                                                                                                                                                                                                       `ctgggaagacgcagaagacttgctcaatgaatacttggaggaagtcgaggaagtcgC‑‑ACGACAGAAAGAAATTGACCGCGCTAAGGCCCGTAAAGAACTTCACGAGGGGCGCTTAGAGGCACGCAGATTCAAACGTCGCAACCGCAAGGCACGTAAAGCACACAAAGCTAAGCGCGAAAGAATGCTTGCTGCGTGGCGATGGGCTGAACGTCAAGAACGGCGTAACCATGCTGTAGCTGTAGATTTACTAGGAAGAACCAATAACGCTATGCTCTGTGTCAACATGTTCCCTGGGGACttt                                                          >  1:81655‑M2/57‑301 (MQ=255)`  
                                                                                                                                                                                                                                         `gggaagacgcagaagacttgctcaatgaatacttggaggaagtcgaggaagtcgC‑‑ACGACAGAAAGAAATTGACCGCGCTAAGGCCCGTAAAGAACGTCACGAGGGGCGCTTAGAGGCACGCAGATTCAAACGTCGCAACCGCAAGGCACGTAAAGCACACAAAGCTAAGCGCGAAAGAATGCTTGCTGCGTGGCGATGGGCTGAACGTCAAGAACGGCGTAACCATGAGGTAGCTGTAGATGTACTAGGAAGAACCAATAACGCTATGCTCTGGGTCAACATGTTCTCTGGGGACTTTaa                                                        >  1:373737‑M2/55‑301 (MQ=255)`  
                                                                                                                                                                                                                                           `gacctctgggaagacgcagaagacttgctcaatgaatacttggaggaagtcgC‑‑ACGACAGAAAGAAATTGACCGCGCTAAGGCCCGTAAAGAACGTCACGAGGGGCGCTTAGAGGCACGCAGATTCAAACGTCGCAACCGCAAGGCACGTAAAGCACACAAAGCTAAGCGCGAAAGAATGCTTGCTGCGTGGCGATGGGCTGAACGTCAAGAACGGCGTAACCATGAGGTAGCTGTAGATGTACTAGGAAGAACCAATAACGCTATGCTCTGGGCCAACATGTTCTCTGGGGACTTTAAgg                                                      <  1:187166‑M2/249‑1 (MQ=255)`  
                                                                                                                                                                                                                                            `aagacgcagaagacttgctcaatgaatacttggaggaagtcgaggaagtcgC‑‑ACGACAGAAAGAAATTGACCGCGATAAGGCCCGTAAAGAACGGCACGAGGGGCGCTTAGAGGCACGCAGATTCAAACGTCGCAACCGCAAGGCACGTAAAGCACACAAAGCTAAGCGCGAAAGAATGCTTGCTGCGTGGCGATGGGCTGAACGTCAAGAACGGCGTAACCATGAGGTAGCTGTAGATGTACTAGGAAGAACCAATAACGCTATGCTCTGGGTCAACATGTTCTCTGGGGACTTTAAGgc                                                     <  1:153246‑M2/250‑1 (MQ=255)`  
                                                                                                                                                                                                                                                        `gtataagagacaggataaatgaatactgggaggaagtcgA‑‑ACGACAGAAAGAAATTGACCGCGCTAAGGCCCGTAAAGAACGTCACGAGGGGCGCTTAGAGGCACGCAGATTCAAACGTCGCAACCGCAAGGCACGTAAAGCACACAAAGCTAAGCGCGAAAGAATGCTTGCTGCGTGGCGATGGGCTGAACGTCAAGAACGGCGTAACCATGAGGTAGCTGTAGATGTACTAGGAAGAACCAATAACGCTATGCTCTGGGTCAACATGTTCTCTGGGGACTTTAAGGCGCTTGAGGAACg                                         <  1:118669‑M2/262‑1 (MQ=255)`  
                                                                                                                                                                                                                                                        `acgcagaagacttgctcaatgaatacttggaggaagtcgC‑‑ACGACAGAAAGAAATTGACCGCGCTAAGGCCCGTAAAGAACGTCACGAGGGGCGCTTAGAGGCACGCAGATTCAAACGTCGCAACCGCAAGGCACGTGAAGCACACAAAGCTAAGAGCGAAAGAATGCTTGCTGCGTGGCGATGGGCTGAACGTCAAGAACGGCGTAACCATGAGGGAGCTGTAGATGTACTAGGAAGAACCAATAACGCTATGCTCTGGGTCAACATGTTATATGGGGACTTTAAGGCGTTTGAGAAACg                                         >  1:294396‑M2/40‑301 (MQ=255)`  
                                                                                                                                                                                                                                                           `cagaagacttgctcaatgaatacttggaggaagtcgC‑‑ACGACAGAAAGAAATTGACCGCGCTAAGGCCCGTAAAGAACGTCACGAGGGGCGCTTAGAGGCACGCAGATTCAAACGTCGCGACCGCAAGGCACGTAAAGCACACAAAGCTAAGCGCGAAAGAATGCTTGCTGCGTGGCGATGGGCTGAACGTCAAGAACGGCGTAACCATGAGGTAGCTGTAGATGTACTAGGAAGAACCAATAACGCTATGCTCTGGGTCAACATGTTCTCTGGGGACTTTAAGGCGCTTGAGgaatgaat                                      >  1:138899‑M2/37‑301 (MQ=255)`  
                                                                                                                                                                                                                                                                    `tgctcaatgaatacttggaggaagtcgC‑‑ACGACAGAAAGAAATTGACCGCGCTAAGGCCCGTAAAGAACGTCACGAGGGGCGCTTAGAGGCACGCAGATTCAAACGTCGCAACCGCAAGGCACGTAAAGCACACAAAGCTAAGCGCGAAAGAATGCTTGCTGCGTGGCGATGGGCTGAACGTCAAGAACGGCGTAACCATGAGGTAGCTGTAGATGTACTAGGAAGAACCAATAACGCTATGCTCTGGGTCaacatgttctctggggactttaaggcctgtctcttatacacatctccgag                             >  1:340833‑M2/28‑277 (MQ=255)`  
                                                                                                                                                                                                                                                                     `gctcaatgaatacttggaggaagtcgC‑‑ACGACAGAAAGAAATTGACCGCGCTAAGGCCCGTAAAGAACGTCACGAGGGGCGCTTAGAGGCACGCAGATTCAAACGTCGCAACCGCAAGGCACGTAAAGCACACAAAGCTAAGCGCGAAAGAATGCTTGCTGCGTGGCGATGGGCTGAACGTCAAGAACGGCGTAACCATGAGGTAGCTGTAGATGTACTAGGAAGAACCAATAACGCTATGCTCTGGGTCAACATGTTCTCTGGGGACTTTAAGGCGCTTGAGGAACGAATCGCGCTGCAc                            >  1:584808‑M2/27‑301 (MQ=255)`  
                                                                                                                                                                                                                                                                        `caatgaatacttggaggaagtcgC‑‑ACGACAGAAAGAAATTGACCGCGCTAAGGCCCGTAAAGAACGTCACGAGGGGCGCTTAGAGTCACGCAGATTCAAACGTCGCAACCGCAAGGCACGTAAAGCACACAAAGCTAAGCGCGAAAGAATGCTTGCTGCGTGGCGATGGGCTAAACGTCAAGAACGGCGTAACCATGAGGTAGCTGTAGATGTACTAGGAAGAACCAATAACGCTATGCTCTGGGTCAACATGTTCTCTGGGGACTTTAAGGCGCTTGAGGAACGAATCGCGCTGCACTgg                         >  1:335783‑M2/24‑301 (MQ=255)`  
                                                                                                                                                                                                                                                                          `atgaatacttggaggaagtcgC‑‑ACGACAGAAAGAAATTGACCGCGCTAAGGCCCGTAAAGAACGTCACGAGGGGCGCTTAGAGGCACGCAGATTCAAACGTCGCAACCGCAAGGCACGTAAAGCACACAAAGCTAAGCGCGACAGAATGCTTGCTGAGTGGCGATGGGCTGAACGTCAAGAACGGCGTAACCATGAGGTAGCTGTAGATGTACTAGGAAGAACCAATAACGCTATGCTCTGGGTCAACATGTTCTCTGGGGCCTTTAAGGCGCTTGAGGAACGAATCGCGCTGCACTGGCg                       >  1:204844‑M2/22‑301 (MQ=255)`  
                                                                                                                                                                                                                                                                                `acttggaggaagtcgC‑‑ACGACAGAAAGAAATTGACCGCGCTAAGGCCCGTAAAGAACGTCACGAGGGGCGCTTAGAGGCACGCAGATTCAAACGTCGCAACCGCAAGGCACGTAAAGCACACAAAGCTAAGCGCGAAAGAATGCTTGCTGCGTGGCGATGGGCTGAACGTCAAGAACGGCGTAACCATGAGGTAGCTGTAGGTGTACTAGGAAGAACCAATAACGCTCTGCTCTGGGTCAACATGTTCTATGGGGACTTTAAGGCGCTTGAGGAACGAATCGCGCTGCACTGTCGTACTGc                 >  1:369391‑M2/16‑301 (MQ=255)`  
                                                                                                                                                                                                                                                                                 `cttggaggaagtcgC‑‑ACGACAGAAAGAAATTGACCGCGCTAAGGCCCGTAAAGAACGTCACGAGGGGCGCTTAGAGGCACGCAGATTCAAACGTCGCAACCGCAAGGCACGTAAAGCACACAAAGCTAAGCGCGAAAGAATGCTTGCTGCGTGGCGATGGGCTGAACGTCAAGAACGGCGTAACCATGAGGTAGCTGTAGATGTACTAGGAAGAACCAATAACGCTATGCTCTGGGTCAACATGTTCTCTGGGGACTTTAAGGCGATTGAGGAACGAATCGCGCTGCACTGGCGTAATGCt                >  1:537649‑M2/15‑301 (MQ=255)`  
                                                                                                                                                                                                                                                                                  `gtcgaggaagtcgC‑‑ACGACAGAAAGAAATTGACCGCGCTAAGGCCCGTAAAGAACGTCACGAGGGGCGCTTAGAGGCACGCAGATTCAAACGTCGCAACCGCAAGGCACGTAAAGCACACAAAGCTAAGCGCGAAAGAATGCTTGCTGCGTGGCGATGGGCTGAACGTCAAGAACGGCGTAACCATGAGGTAGCTGTAGATGTACTAGGAAGAACCAATAACGCTATGCCCTGGGTCAACATGTTCTCTGGGGACTTTAAGGCGCTTGAGGAACGAATCGCGCTGCACTGGCGTAATGCtt               >  1:604527/1‑300 (MQ=255)`  

                                                                                                                                                                                                                                                                                               `|`                                                                                                                                                                                                                                                                                                                
`CAATGATGCCCATTATAAATACGCTGAGATTGCAAGCGACATCATTGATTGCAATTCGGATGAGCATGATGAGTTAACTGGATGGGATGGTGAGTTTGTTGAAACTTGTAAACTAATCCGCAAGTTCTTTGAGGGCATCGCCTCATTCGACATGCATAGCGGGAACATCATGTTCTCAAATGGAGACGTACCATACATCACCGACCCGGTATCATTCTCGCAGAAGAAAGACGGTGGCGCATTCAGCATCGACCCTGAGGAACTCATCAAGGAAGTCGAGGAAGTCGC‑‑ACGACAGAAAGAAATTGACCGCGCTAAGGCCCGTAAAGAACGTCACGAGGGGCGCTTAGAGGCACGCAGATTCAAACGTCGCAACCGCAAGGCACGTAAAGCACACAAAGCTAAGCGCGAAAGAATGCTTGCTGCGTGGCGATGGGCTGAACGTCAAGAACGGCGTAACCATGAGGTAGCTGTAGATGTACTAGGAAGAACCAATAACGCTATGCTCTGGGTCAACATGTTCTCTGGGGACTTTAAGGCGCTTGAGGAACGAATCGCGCTGCACTGGCGTAATGCTGACCGGATGGCTAT  >  NC_001604.1/2455‑3042`

  

Alignment Legend

Aligned base mismatch/match (shaded by quality score): `ATCG/ATCG` `< 3 ≤` `ATCG/ATCG` `< 10 ≤` `ATCG/ATCG` `< 22 ≤` `ATCG/ATCG` `< 34 ≤` `ATCG/ATCG` `< 38 ≤` `ATCG/ATCG`

Unaligned base: `atcg`    Masked matching base: `atcg`    Alignment gap: `‑`    Deleted base: `‑`
